# Development of SYBR green I-based real-time qPCR differential diagnosis assays for porcine reproductive and respiratory syndrome virus typing in Guangdong province

**DOI:** 10.3389/fvets.2025.1495128

**Published:** 2025-03-05

**Authors:** Zhaowen Ren, Pu Kang, Pian Zhang, Chenglong Sun, Jing Chen, Hua Xiang, Shengjun Luo, Rujian Cai, Yuan Huang, Yuzhu Jin, Gang Wang, Xiaohu Wang

**Affiliations:** ^1^Guangdong Province Key Laboratory of Livestock Disease Prevention, Key Laboratory for Prevention and Control of Avian Influenza and Other Major Poultry Diseases, Ministry of Agriculture and Rural Affairs, Institute of Animal Health, Guangdong Academy of Agricultural Sciences, Guangzhou, China; ^2^Guangdong Provincial Key Laboratory of Zoonosis Prevention and Control, College of Veterinary Medicine, South China Agricultural University, Guangzhou, China; ^3^College of Life Science and Engineering, Foshan University, Foshan, China; ^4^Department of Molecular Biology, University of Texas Southwestern Medical Center, Dallas, TX, United States; ^5^Jiaozuo City Product Quality Inspection and Testing Center, Jiaozuo, China

**Keywords:** PRRSV, duplex real-time PCR, prevalence, phylogenetic analysis, differential diagnosis

## Abstract

**Introduction:**

Porcine Reproductive and Respiratory Syndrome (PRRS) is a highly contagious disease that causes reproductive disorders in sows and respiratory problems in pigs of different ages. It first appeared in the late 20th century in the United States and Europe before spreading globally, leading to significant economic losses in the swine industry. Porcine Reproductive and Respiratory Syndrome virus (PRRSV) has a high rate of genetic recombination, resulting in considerable genetic diversity within the virus. The lack of cross-protection between different lineages often leads to unsuccessful vaccination attempts.

**Methods:**

To accurately distinguish PRRSV lineages and develop effective vaccination strategies for pigs, we have developed a fluorescence quantitative PCR (qPCR) method by designing specific primers and SYBR green dye. This method allows for the simultaneous identification of different PRRSV genotypes.

**Results:**

Our experimental results show that these methods have good specificity and do not react with other common viral pathogens in pigs. This method also demonstrates good sensitivity, with the ability to detect low levels of the virus. The detection limits of these assay were 10^2^ copies/μL for PRRSV-1 (European-type PRRS) and 10^1^ copies/μL for PRRSV-2 (American-type PRRSV), HP-PRRSV (Highly Pathogenic PRRSV), and NL-PRRSV (NADC30-like PRRSV), respectively. Furthermore, the reproducibility of this method is commendable, with intra- and inter-assay coefficients of variation remaining below 3%. In the subsequent study, a total of 316 clinical samples of porcine with respiratory and reproductive failure symptoms were collected from 14 cities in Guangdong. The results showed that among these samples, 22.78% (72 out of 316) tested positive for PRRSV-2, 15.51% (49 out of 316) tested positive for HP-PRRSV, and 0.95% (3 out of 316) tested positive for NL-PRRSV. However, PRRSV-1 was not detected in any of the samples.

**Discussion:**

Our method provides a quick way to identify PRRSV genotypes in pig herds in Guangdong, which has certain significance for developing effective vaccination strategies against PRRS.

## 1 Introduction

The Porcine Reproductive and Respiratory Syndrome (PRRS) is a highly contagious infectious disease caused by the Porcine Reproductive and Respiratory Syndrome virus (PRRSV). It has had a significant negative impact on the global swine industry for the past 30 years, resulting in billions of dollars in losses (1). The PRRSV genome is approximately 15 kb in size and consists of at least 11 open reading frames responsible for encoding over 16 structural and non-structural proteins (2). Among all the proteins encoded by PRRSV, NSP2 and GP5 exhibit the highest variability. NSP2, with approximately 980 amino acids (aa), is a large protein that displays significant genetic diversity, with around 40% aa homology between PRRSV-1 (European-type PRRS) and PRRSV-2 (American-type PRRSV). Compared to the PRRSV-2 prototype strain (VR-2332, lineage 5), NSP2 of other circulating strains in China exhibit aa deletions at different sites. Specifically, the HP-PRRSV (Highly Pathogenic PRRSV) strain has discontinuous aa deletions at positions 482 and 532–560, totaling 1 + 29 aa. The NADC30 strain (lineage 1.8) has 111 + 1 + 19 discontinuous aa deletions at positions 324–434, 482, and 505–523. Both NADC34 and PRRSV 1–4-4 strains have a deletion of 100 aa at positions 330–429.

The virus was first identified in North America in 1987 and later spread to China in 1996 (3). Initially, classical strains like CH-1a (GenBank: AY032626) and BJ-4 (GenBank: AF331831) were predominant in Chinese swine herds for about a decade. However, in 2006, a highly pathogenic variant called HP-PRRSV emerged in China and replaced the CH-1a strain as the dominant strain (4). In 2014, another strain known as NL-PRRSV (NADC30-like PRRSV) was identified in China. This strain exhibits extensive recombination with different lineages, resulting in increased genetic diversity and variable levels of virulence, making it highly adaptable (5, 6). This led to its rapid prevalence within Chinese pig herds (7). At present, the NL-PRRSV and HP-PRRSV strains continue to pose significant threats to pig herds across the majority of China’s regions. However, since 2017, there has been an increasing positive rate for the NADC34-like strain, indicating its potential emergence as the new predominant strain (8).

Vaccination stands as a fundamental strategy in the control of PRRSV infection. However, due to the limited cross-protection of vaccines against different strains of the virus, immunization failures are common. Therefore, to achieve precise control of PRRSV, obtaining timely genetic information on the prevailing strains in pig herds is crucial. Previous studies have shown that amplification of the ORF5 segment of the virus by RT-PCR, followed by phylogenetic analysis, is a major molecular biology approach for PRRSV genotyping (9–11). Yet, this technique is intricate and time-intensive, and RT-PCR amplification of long fragments is less sensitive and often difficult. To circumvent such limitations, Yang et al. developed a multiplex RT-PCR method based on a single RT-PCR to differentiate HP-PRRSV, HP-PRRSV vaccine strains, and classical PRRSV (12). However, sensitivity and specificity issues still exist. In last two decades, with the development of fluorescence labeling techniques, Wen et al. to devise a TaqMan probe-based fluorescent quantitative PCR technique capable of concomitant identification of American type, European type, and highly pathogenic PRRSV, enhancing detection efficacy and providing a more seamless and precise method for PRRSV genotype identification (13). However, the cost of TaqMan probe-based techniques often render them impractical in primary swine farms. To address these issues, our research has established a qPCR method based on SYBR Green I. This method allows the simultaneous identification of PRRSV-1 and PRRSV-2 genotypes, as well as HP-PRRSV and NL-PRRSV genotypes. This approach effectively distinguishes the four genotypes of PRRSV, while being cost-effective, user-friendly, time-efficient, highly sensitive, and highly specific. Therefore, it is suitable in primary swine farms and laboratories.

## 2 Materials and methods

### 2.1 Primers

A total of 407 complete PRRSV genome sequences from China were downloaded from the GenBank database (Supplementary Table S1). The sequences were aligned using MAFFT (Version 4.470) (14), and conserved regions of the target lineage strains were selected for primer design. Specific primers for different types of strain were designed at the conserved sequences using primer 5.0 software. And the primer melting temperature (Tm), hairpin structures, and complementarity were predicted using Oligo (Version 7) software (15). All primers were synthesized by Beijing Tsingke Biotech Co., Ltd. (Beijing, China). The details of all primers have been listed in Table 1.

**Table 1:** Primers used in this study.

No.	Use	Primers	Sequence (5′ end to 3′ End)	Region
1	Amplification of PRRSV-1 ORF5	EU-ORF5-F	TCTGGCGTTGTTTCTGCTT	13,317–14,275 (16)
		EU-ORF5-R	CGACACGGTTGGTGGATTG	
2	Amplification of NL-PRRSV ORF5	NL-ORF5-F	ACYGTTTTAGCCTGT	13,344–14,058
		NL-ORF5-R	RTATATCATTATTGGCGT	
3	Amplification of PRRSV-2 ORF5	ORF5-F	ACCTGAGACCATGAGGTGGGCAA	13,624–14,397 (17)
		ORF5-R	CGGCCGCGACTTACCTTTAGAGCAT	
4	For detection of PRRSV-1	EU-F	TCCTTGCCATACTGTTTG	11,681–11,723
		EU-R	CACYCTGAGAAGAAAGACCA	
5	For detection of PRRSV-2	US-F	CTCCAGRTGCCGKTTGT	14,572–14,619
		US-R	TCRACGTGGTGGGCAG	
6	For detection of HP-PRRSV	HP-F	GGTTCGGAAGAAACTGTCGG	2,767–2,901
		HP-R	GCGGTGCWGGAACTGGT	
7	For detection of NL-PRRSV	NL-F	CCTGTAACCAARRTTTC	13,954–14,061
		NL-R	AGGCATATATCATTATTGG	

### 2.2 Viruses

For the validation of method specificity, commercially obtained attenuated vaccines of Porcine Epidemic Diarrhea Virus (PEDV, strain AJ1102-R, sourced from Wuhan Keqian Biology Co., Ltd.), Porcine Transmissible Gastroenteritis Virus (TGEV, strain WH-1R, sourced from Wuhan Keqian Biology Co., Ltd.), Porcine Pseudorabies Virus (strain Bartha-K16, acquired from China Animal Husbandry Industry Co., Ltd.), and Porcine Circovirus Type 2 (PCV2, strain WH, supplied by China Animal Husbandry Industry Co., Ltd.) were employed.

### 2.3 DNA/RNA extraction and reverse transcription

Nucleic acids from PRRSV, PEDV, TGEV, PRV, and PCV2 were extracted using the RaPure Viral RNA/DNA Kit (Magen, Guangzhou, China) according to the manufacturer’s instructions. The HiScript® II 1st Strand cDNA Synthesis Kit (Vazyme, Nanjing, China) was then used to synthesize cDNA by reverse transcription. DNA and cDNA products were stored at −80°C.

### 2.4 Recombinant plasmid construction

To construct the recombinant standard plasmid, the target fragments of different PRRSV lineages were amplified by primer pairs 4 to 7 (2× Taq Plus Master Mix II, Vazyme, Nanjing, China). Next, according to the manufacturer’s instructions, the PCR products were purified using the Magen Gel Extraction Kit (Magen, Guangzhou, China). The purified products were cloned into the pMD18-T vector (Takara, Beijing, China) and transformed into DH5α competent cells (Vazyme, Nanjing, China). Then, the bacterial cultures were shaken and grown for 12 h at 37°C, and the final plasmid obtained was named PMD-EU, PMD-US, PMD-HP, and PMD-NL, respectively. The recombinant plasmid was purified using the HiPure Plasmid Micro Kit (Magen, Guangzhou, China) and the concentration was determined using NanoDrop spectrophotometer (Thermo Scientific, United States). Finally, the concentration was converted into a copy number using the following formula:


$$y\left(\mathrm{copies}/\mathrm{\mu L}\right)=\frac{\left(6.02\times 10^{23}\right)\times \left(x\;\mathrm{ng}/\mathrm{\mu L}\times 10^{-9}\right)}{DNA\;\mathrm{length}\times 660}$$

### 2.5 Multiplex qPCR assay optimization

We further optimized the conditions for multiple qPCR, including annealing temperature and primer concentration. The standard plasmids were 10-fold serial diluted from 1 × 10^6^ copies/μL down to 1 × 10^2^ copies/μL as amplification templates. The reaction system was 20 μL, including 10 μL of 2 × ChamQ Universal SYBR qPCR Master Mix (Vazyme, Nanjing, China), 2 μL of standard plasmid as a template, different final concentrations of primers, and nuclease-free water. A matrix approach was then used to explore the optimal reaction conditions for this multiple reaction: annealing temperatures of 50, 55, and 60°C; and final primer concentrations of 100 nM-350 nM at five dilutions. Amplification conditions were pre-denaturation at 95°C for 5 min, followed by 40 cycles of 95°C for 10 s, and annealing between 50°C and 60°C for 30s. Fluorescence signals were collected by a LightCycler® 96 thermal cycler Instrument (Roche Applied Science, Penzberg, Germany). The system was optimized by generating the lowest threshold cycle (Ct) and the highest cycle increase (ΔRn) for each specific fluorescent signal.

Based on the results of the amplification, the optimal annealing temperature was selected, and the proportions of primers numbered 4, 5, and 6, 7, were adjusted until a clear double-peak melting curve appeared and corresponded to good amplification efficiency. The primer proportions at this point were considered the optimal primer proportions.

### 2.6 Standard curve construction

The standard plasmids were continuously diluted tenfold from 1 × 10^6^ copies/μL to 1 × 10^2^ copies/μL. Under optimal reaction conditions and systems, these five concentrations of standard plasmid were used as templates to construct the standard curve. The standard curve was plotted using GraphPad Prism software (version 8).

### 2.7 Specificity, sensitivity, repeatability assay

To evaluate the specificity of multiplex qPCR assay, DNA or cDNA of PEDV, TGEV, PRV and PCV2 extracted from the vaccine strains were used as templates, using standard plasmids PMD-HP, PMD-NL, PMD-EU, and PMD-US as positive controls and ddH_2_O as a negative control, and the specificity was verified with qPCR assay under the optimal reaction condition and system. In order to determine the detection limit of the developed detection method in this study, the standard plasmids were continuously diluted tenfold from 10^6^ copies/μL to 10^0^ copies/μL. And qPCR amplification was performed in the optimum reaction conditions and system. Each concentration was tested in triplicate to eliminate differences caused by technical and operational factors.

Finally, in order to assess the reproducibility of the method, the four varieties of standard plasmids (10^1^ copies/μL to 10^6^ copies/μL) were used as templates to evaluate intra- and inter-assay reproducibility. For intra-assay reproducibility, each dilution was replicated three times daily under identical conditions. For inter-assay reproducibility, according to MIQE guidelines (16), each dilution was tested in six independent experiments performed by two operators on different days. Coefficients of variation of the Ct values were calculated based on the intra-assay or inter-assay results.

### 2.8 Comparison and verification of detection performance

To verify the detection performance of this method, we compared and validated the detection method established in this study with commercial detection kits. Differential diagnosis of PRRSV was performed using the commercial kit VetMAX PRRSV EU&NA 2.0 Kit (Thermo, United States, A35751) according to the manufacturer’s instructions.

For sensitivity comparison experiments, four standard plasmids (PMD-EU, PMD-US, PMD-HP, and PMD-NL) (10^1^ copies/μL to 10^3^ copies/μL) were used as templates for evaluation (*n* = 24 for each plasmid and concentration, and *n* = 24 for negative controls). For specificity comparative experiments, nucleic acids from PRRSV, PEDV, TGEV, PRV, and PCV2 were used as templates for evaluation (*n* = 24 for each virus, and *n* = 24 for negative controls). For reliability comparison tests, we evaluated the nucleic acids of PRRSV-1 (*n* = 15), PRRSV-2 (*n* = 15), HP-PRRSV (*n* = 15), NL-PRRSV (*n* = 15) positive samples, and PRRSV negative samples (*n* = 15) previously stored in our laboratory. It should be noted that because of the low prevalence of PRRSV-1 in mainland China in recent years, we divided the only PRRSV-1 nucleic acid sample into 15 parts to expand the number of positive samples.

Finally, we calculated the agreement rate between our detection method and commercial reagent kits (the total number of true positives and true negatives/the sum of true positives, true negatives, false positives, and false negatives) to demonstrate the detection performance of our constructed method.

### 2.9 Clinical sample detection

**Figure 1:**
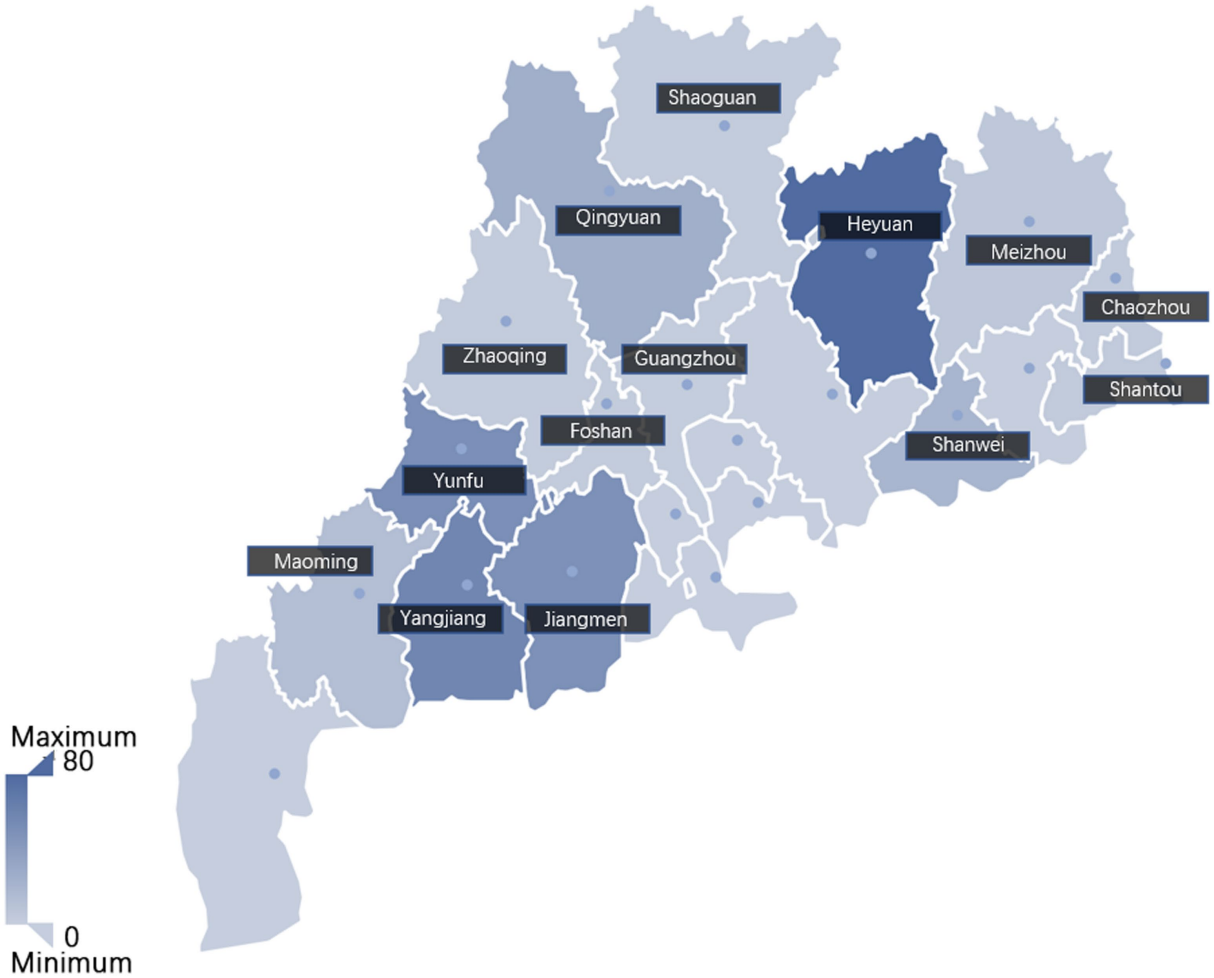
The geographic distribution of the clinical samples analyzed in this study. Sample size from each city was indicated with color depth on the map. The intensity of the color correlates to the number of samples, with darker shades indicating a higher number of samples.

Between 2021 and 2023, a total of 316 clinical samples, including 139 tissues samples, 78 blood samples and 99 serum samples, were collected from swine farms in 14 cities in Guangdong Province, China (Figure 1). The detailed information of the samples was listed in Supplementary Table S2. These samples were subsequently analyzed to determine the infection positivity rate of different lineages using the established multiplex qPCR assay.

### 2.10 The complete ORF5 gene amplification and sequencing

Next, the complete ORF5 gene sequences of representative positive samples were amplified using primer pairs 1, 2 and 3 from Table 1. The reaction system was 50 μL, containing 25 μL of 2 × KeyPo SE Master Mix (Dye Plus) (Vazyme, Nanjing, China), 2 μL of template cDNA, 10 μM forward and reverse primers with 2 μL each, and the remaining was added to ddH_2_O. The PCR reaction for ORF5 gene was executed by pre-denaturation at 94°C for 2 min, followed by 35 cycles of 98°C for 10 s, 55°C (other PRRSV strains) or 45°C (NL-PRRSV) for 30 s, and 68°C for 15 s. The PCR products were then purified using the Magen Gel DNA Recovery Kit (Magen, Guangzhou, China) according the manufacturer’s instructions. The purified PCR products were cloned using a pGM-T cloning kit (Tiangen, Beijing, China), and propagated in DH5α competent cells (Vazyme, Nanjing, China) according to the manufacturer’s instructions. Positive clones were sequenced by Beijing Tsingke Biotech Co., Ltd. The obtained complete ORF5 gene sequences were edited and assembled using DNAstar V7.1 software.

### 2.11 Phylogenetic analysis

In order to further investigate the genetic characteristics of PRRSV in the surveyed regions of Guangdong Province, a total of 13 complete ORF5 gene sequences of representative PRRSV strains were obtained in this study and uploaded to NCBI GenBank with accession numbers OR539223-OR539235. The complete ORF5 gene sequences of PRRSV were aligned with the relevant reference sequences in GenBank using MAFFT version 7.487 with the parameter L-INS-I. Phylogenetic trees were generated by the neighbor-joining (NJ) method in MEGA X with the bootstrap of 1,000 replicates. Details of the reference sequence were listed in Supplementary Table S3.

### 2.12 Data–statistical analysis

In the specificity and repeatability tests, we conducted three tests on each sample, and the final results were expressed as mean ± standard deviation. In the repeatability test, we determined the differences within and between groups by calculating the coefficient of variation (CV). In addition, the data and statistical analyses of PRRSV prevalence were estimated from the ratio of positive samples to the total number of samples analyzed, with a binomial confidence interval of 95%. The data were analyzed using Microsoft Excel 2019 and SPSS version 27.0.

## 3 Results

### 3.1 Optimization of qPCR reaction conditions

Annealing temperatures of 50, 55, and 60°C, and primer final concentrations of 100–350 nM were selected for the two types of duplex qPCR system optimization. According to the optimization results, the annealing temperature was determined to be 55°C for NL-PRRSV and HP-PRRSV (Supplementary Table S4), 50°C for PRRSV-1 and PRRSV-2 (Supplementary Table S5). The final primer concentrations for NL-PRRSV, HP-PRRSV, PRRSV-1, and PRRSV-2 were 250, 250, 200, and 300 nM, respectively (Table 2).

**Table 2:** Optimization of primer concentrations of duplex qPCR assay.

Primer concenteation (nM)	Template	Plasmid concentration(copies/μL)					Amplification efficiency (%)
		1 × 10^6^	1 × 10^5^	1 × 10^4^	1 × 10^3^	1 × 10^2^	
100	NL-PRRSV	17.09	20.64	24.31	27.77	32.40	84.03
	HP-PRRSV	15.81	19.42	23.13	26.83	30.69	85.78
	PRRSV-1	20.67	24.25	27.51	31.15	33.03	107.11
	PRRSV-2	18.91	23.21	27.53	31.89	35.30	74.26
150	NL-PRRSV	16.11	19.47	22.67	26.38	30.60	89.93
	HP-PRRSV	15.27	18.75	22.17	25.63	29.18	94.18
	PRRSV-1	20.57	23.92	27.13	30.90	34.47	93.87
	PRRSV-2	17.09	20.67	24.49	28.50	31.92	84.80
200	NL-PRRSV	15.80	18.66	22.22	25.47	29.46	96.34
	HP-PRRSV	15.00	18.52	21.88	24.76	28.54	99.57
	PRRSV-1	20.46	23.63	27.08	30.42	33.95	97.74
	PRRSV-2	16.29	19.76	23.25	27.01	30.24	92.53
250	NL-PRRSV	15.41	18.72	21.97	25.19	29.08	97.61
	HP-PRRSV	15.25	18.52	21.82	25.10	28.61	99.66
	PRRSV-1	20.57	23.94	27.06	30.86	35.41	87.59
	PRRSV-2	15.95	19.33	22.78	26.48	29.68	94.49
300	NL-PRRSV	15.17	18.15	21.55	25.01	28.97	95.07
	HP-PRRSV	15.05	18.52	21.86	25.22	28.51	98.34
	PRRSV-1	20.39	23.58	26.90	30.78	35.56	84.67
	PRRSV-2	15.71	19.02	22.47	26.06	29.11	97.47
350	NL-PRRSV	15.06	18.04	21.42	24.89	28.78	95.70
	HP-PRRSV	15.35	18.66	21.91	25.18	28.50	101.70
	PRRSV-1	20.37	23.54	26.89	30.75	35.43	85.31
	PRRSV-2	15.72	18.87	22.34	26.03	29.18	96.52

After optimization, the two types of duplex qPCR enable simultaneous detection of all target nucleic acids. Consequently, the finalized amplification systems for the two types of duplex-qPCRs were established as follows: for NL-PRRSV and HP-PRRSV, 10 μL of 2 × ChamQ Universal SYBR qPCR Master Mix, 250 nM HP-PRRSV primer, and 250 nM NL-PRRSV primer, 2 μL of template, the remaining volume was made up to 20 μL with ddH_2_O. For PRRSV-1 and PRRSV-2, 10 μL of 2 × ChamQ Universal SYBR qPCR Master Mix, 200 nM PRRSV-1 primer, and 300 nM PRRSV-2 primer, 2 μL of template, the remaining volume was made up to 20 μL with ddH_2_O.

The optimal reaction conditions were pre-denaturation at 95°C for 10 s, followed by 40 cycles of 95°C for 10 s and 50°C (PRRSV-1 and PRRSV-2) or 55°C (NL-PRRSV and HP-PRRSV) for 30 s. The fluorescence signals were captured with a real-time qPCR instrument from Roche for the entire duration of the study.

According to the calculations by oligo 7.0 software, the expected Tm values of the primer pairs used for differential diagnosis in this study were 79°C (PRRSV-1), 84°C (PRRSV-2), 84°C (NL-PRRSV), and 88°C (HP-PRRSV), respectively. These values were used to distinguish PRRSV-1, PRRSV-2, NL-PRRSV, and HP-PRRSV. It is worth noting that in actual testing, there is a deviation between the Tm values and the expected values. The actual Tm values (mean ± SD) were as follows: 79.71 ± 0.23°C (PRRSV-1), 84.43 ± 0.12°C (PRRSV-2), 84.34 ± 0.26°C (NL-PRRSV), and 87.86 ± 0.13°C (HP-PRRSV), respectively.

### 3.2 Duplex qPCR assay standard curve

**Figure 2:**
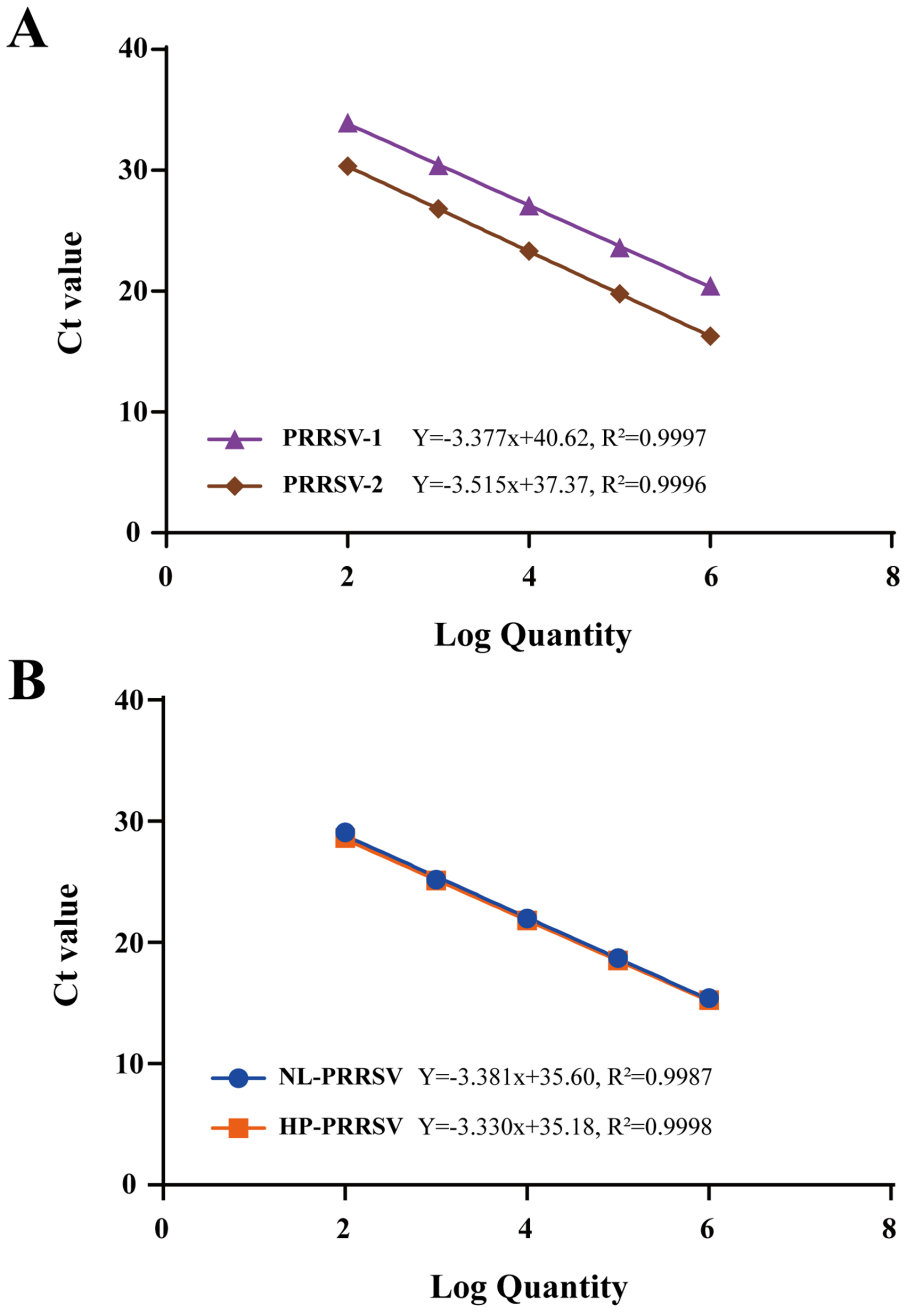
Standard curves of duplex qPCR assay. **(A)** PRRSV-1/PRRSV-2 duplex qPCR Standard Curve. **(B)** HP-PRRSV/NL-PRRSV duplex qPCR Standard Curve. Serial 10-fold dilutions of the standard plasmid were generated at final concentrations of 1 × 10^6^–1 × 10^2^ copies/μL. Data are from three independent studies. The threshold cycles (Ct) from the duplex qPCR assay are plotted against the log numbers of the standards. Mean ± standard deviations are shown for each individual study.

The recombinant standard plasmid, diluted to 1 × 10^6^ to 1 × 10^2^ copies/μL in a 10-fold gradient, was amplified using the two types of duplex qPCR assay according to the optimal reaction system and reaction procedure. The standard curve was established using obtained Ct values as the y axis coordinates and the logarithm of the plasmid concentration as the x axis coordinates. All standard curves had good correlation coefficients and amplification efficiencies. The slopes, correlation coefficient (*R*^2^), and amplification efficiency (Eff%) were as follows: NL-PRRSV (−3.381, *R*^2^ = 0.9987, and Eff% = 97.59), HP-PRRSV (−3.330, *R*^2^ = 0.9998, and Eff% = 99.66), PRRSV-1 (−3.377, *R*^2^ = 0.9997, and Eff% = 97.75), and PRRSV-2 (−3.515, *R*^2^ = 0.9996, and Eff% = 92.53) (Figure 2).

### 3.3 Duplex qPCR assay sensitivity

**Figure 3:**
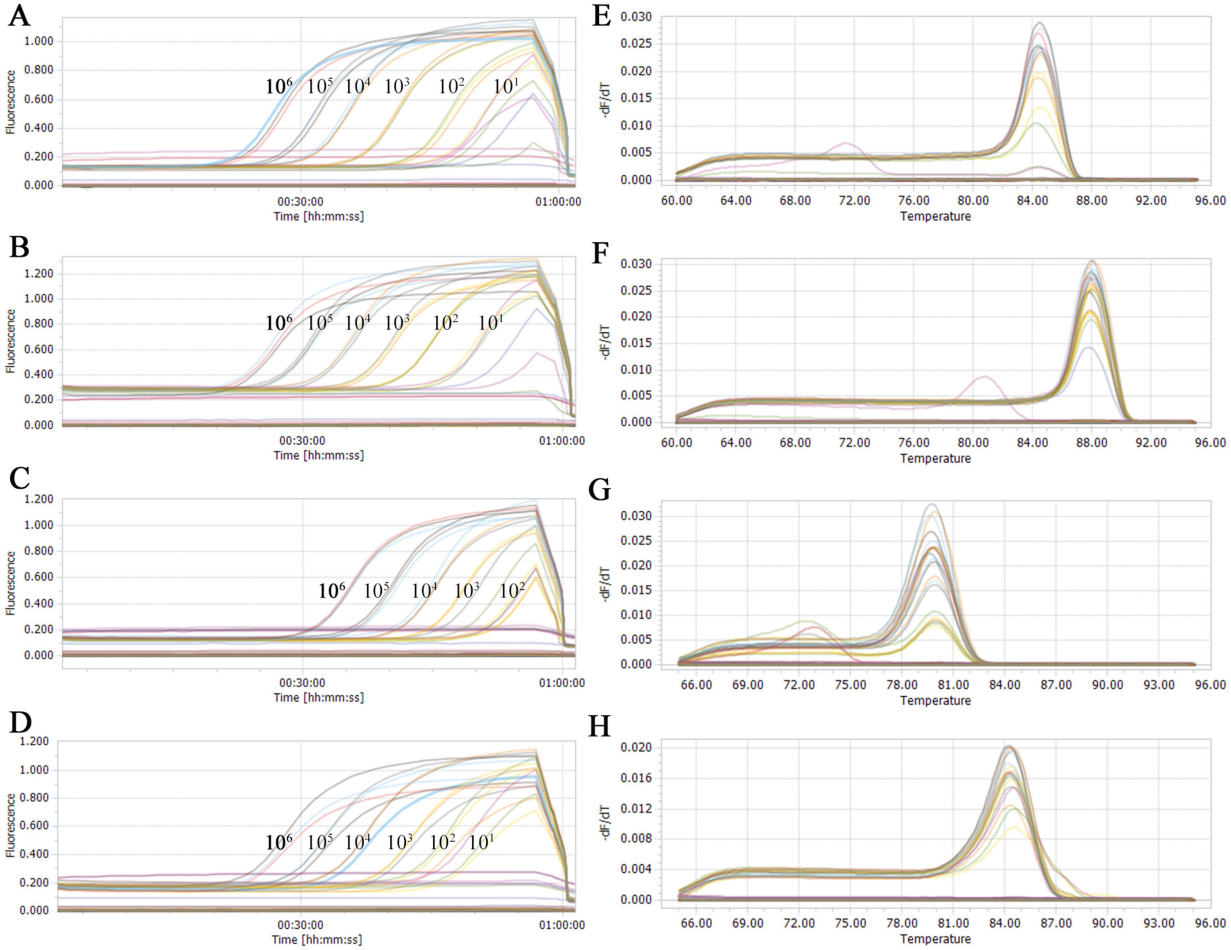
Sensitivity analysis of double qPCR assay. Serial 10-fold dilutions of the standard plasmid were generated at final concentrations of 1 × 10^6^ to 1 × 10^0^ copies/μL. The qPCR assays have the detection limit at 1 × 10^2^ (PRRSV-1) or 1 × 10^1^ (PRRSV-2, HP-PRRSV, and NL-PRRSV) copies/μL for each types of PRRSV. **(A)** Sensitivity analysis of NL-PRRSV. **(B)** Sensitivity analysis of HP-PRRSV. **(C)** Sensitivity analysis of PRRSV-1. **(D)** Sensitivity analysis of PRRSV-2. **(E)** Melting curves of NL-PRRSV. **(F)** Melting curve of HP-PRRSV. **(G)** Melting curves of PRRSV-1. **(H)** Melting curves of PRRSV-2.

To assess the sensitivity of our developed qPCR assay method, the two types of duplex qPCR were performed using standard plasmids with concentrations ranging from 1 × 10^6^ to 1 × 10^0^ copies/μL. The results showed that the established detection limit for PRRSV-1 was determined to be 10^2^ copies/μL (Figure 3C), and the detection limits for NL-PRRSV (Figure 3A), HP-PRRSV (Figure 3B), and PRRSV-2 (Figure 3D) were ascertained to be 10^1^ copies/μL, suggesting that our duplex qPCR methods have a good sensitivity.

Furthermore, clear distinctions between HP-PRRSV (Figure 3E) and NL-PRRSV (Figure 3F), as well as between PRRSV-1 (Figure 3G) and PRRSV-2 (Figure 3H), were observable based on the derived melting curves. In conclusion, we suggested that both duplex SYBR Green qPCR assay methods demonstrated excellent detection and discrimination capabilities.

### 3.4 Duplex qPCR assay specificity

**Figure 4:**
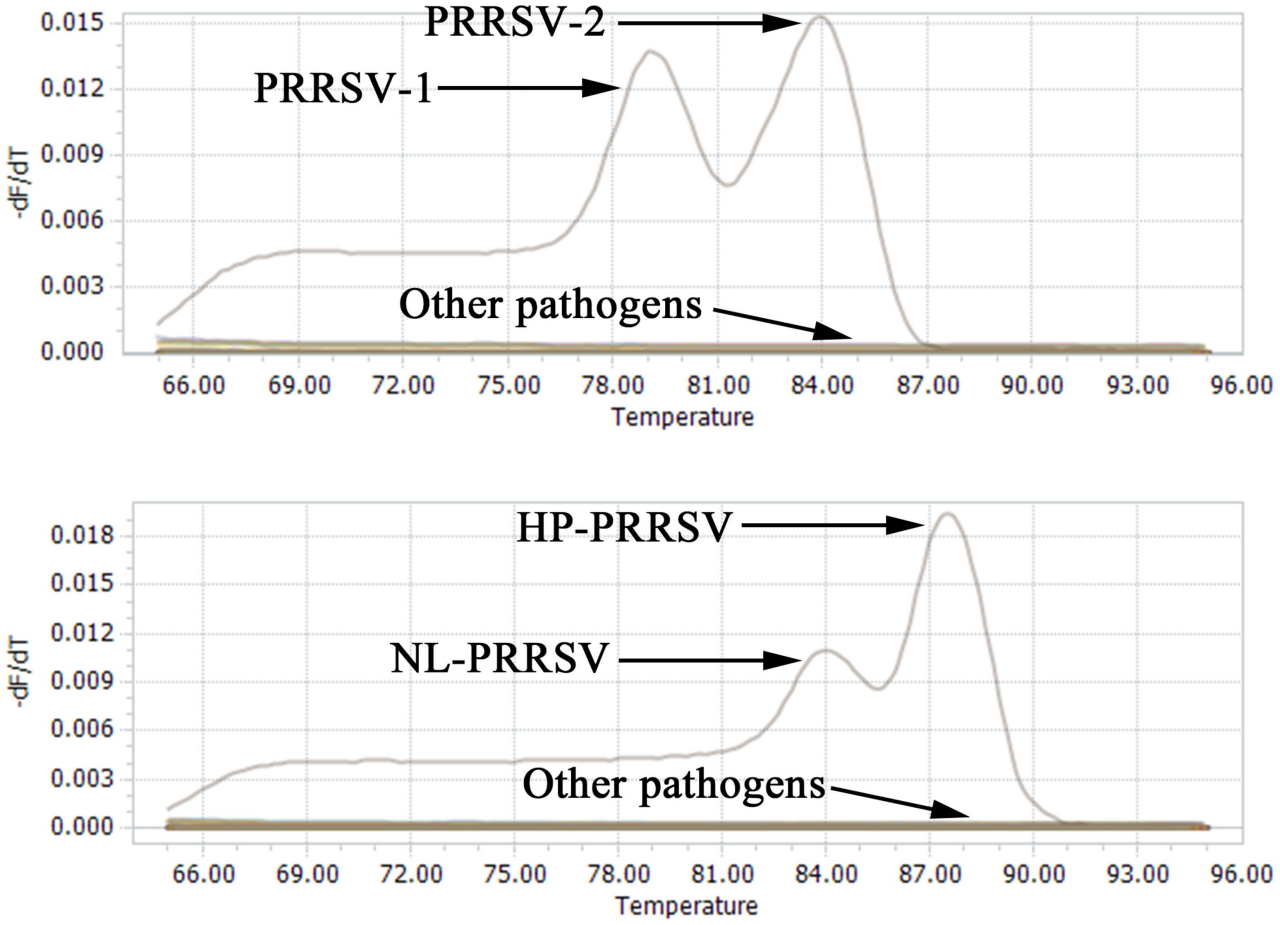
Specificity analysis of double qPCR assay. When PMD-EU, PMD-US, PMD-HP, and PMD-NL plasmid were used, fluorescent signals were specifically detected, respectively. No specific fluorescence signal was obtained when testing other viruses (PEDV, TGEV, PRV and PCV2) and negative controls.

To further assess the specificity of the duplex qPCR assay methods, the recombinant standard plasmids PMD-EU, PMD-US, PMD-HP, and PMD-NL were employed as positive controls. DNA or cDNA extracted from PEDV, TGEV, PRV and PCV2 vaccines were used as templates, and ddH_2_O was used as a negative control. SYBR Green qPCR was performed using the optimal conditions of amplification. The outcomes demonstrated that this technique specifically identified the nucleic acids of PRRSV-1, PRRSV-2, HP-PRRSV, and NL-PRRSV exclusively in the positive control plasmids (Figure 4), with no amplification detected for the other viral pathogens (Table 3). These results suggest that our identification assays are highly specific.

**Table 3:** CT values and melting peaks of specificity analysis of duplex qPCR.

Pathogen	CT Value	Melting peaks
NL-PRRSV	17.40 ± 0.16	84°C
HP-PRRSV	17.40 ± 0.16	88°C
PRRSV-1	18.47 ± 0.11	79°C
PRRSV-2	18.47 ± 0.11	84°C
PEDV	–	–
TEGV	–	–
PRV	–	–
PCV2	–	–

### 3.5 Duplex qPCR assay repeatability

The repeatability and reproducibility of the constructed duplex SYBR Green qPCR assay were assessed using the recombinant standard plasmids, which were diluted in a 10-fold gradient from 10^6^ copies/μL to 10^1^ copies/μL as templates. As shown in Table 4, the results revealed that the coefficient of variation (CV) for both intra-assay and inter-assay replicates of the CT values ranged from 0.02 to 2.12% and 0.02 to 2.28%, respectively. These results indicate that our qPCR assay established in this study has good reproducibility and reliability.

**Table 4:** Repeatability and reproducibility analyses of duplex qPCR assay.

Template	Plasmid concentration(copies/μL)	Intra reproductivity		Inter reproductivity	
		Mean ± S.D.	CV (%)	Mean ± S.D.	CV (%)
PRRSV-1	1 × 10^6^	20.93 ± 0.190	0.91%	20.79 ± 0.150	0.72%
	1 × 10^5^	24.25 ± 0.060	0.25%	24.22 ± 0.060	0.25%
	1 × 10^4^	27.80 ± 0.005	0.02%	27.82 ± 0.130	0.47%
	1 × 10^3^	31.50 ± 0.170	0.54%	31.04 ± 0.290	0.93%
	1 × 10^2^	35.44 ± 0.200	0.56%	33.82 ± 0.280	0.83%
	1 × 10^1^	–	–	–	–
PRRSV-2	1 × 10^6^	15.63 ± 0.060	0.38%	15.78 ± 0.080	0.51%
	1 × 10^5^	18.99 ± 0.030	0.16%	19.02 ± 0.060	0.32%
	1 × 10^4^	22.41 ± 0.020	0.09%	22.46 ± 0.060	0.27%
	1 × 10^3^	26.03 ± 0.080	0.31%	26.04 ± 0.040	0.15%
	1 × 10^2^	29.17 ± 0.050	0.17%	29.16 ± 0.120	0.41%
	1 × 10^1^	31.46 ± 0.260	0.83%	31.77 ± 0.090	0.28%
HP-PRRSV	1 × 10^6^	15.23 ± 0.100	0.65%	15.11 ± 0.010	0.07%
	1 × 10^5^	18.41 ± 0.020	0.11%	18.34 ± 0.110	0.60%
	1 × 10^4^	21.78 ± 0.050	0.23%	21.75 ± 0.090	0.41%
	1 × 10^3^	25.04 ± 0.040	0.16%	24.99 ± 0.090	0.36%
	1 × 10^2^	28.43 ± 0.090	0.32%	28.45 ± 0.060	0.21%
	1 × 10^1^	32.16 ± 0.380	1.18%	31.95 ± 0.230	0.72%
NL-PRRSV	1 × 10^6^	15.13 ± 0.320	2.12%	15.36 ± 0.110	0.72%
	1 × 10^5^	18.31 ± 0.190	1.04%	18.11 ± 0.290	1.60%
	1 × 10^4^	20.97 ± 0.140	0.67%	21.03 ± 0.480	2.28%
	1 × 10^3^	24.89 ± 0.030	0.12%	24.88 ± 0.005	0.02%
	1 × 10^2^	29.39 ± 0.280	0.95%	29.25 ± 0.040	0.14%
	1 × 10^1^	32.26 ± 0.210	0.65%	32.58 ± 0.400	1.23%

### 3.6 Comparison of duplex qPCR assay performance

By comparing with commercially available reagent kits, this study further validated the detection performance of the established duplex qPCR method, including sensitivity, specificity, and effectiveness. The results showed that when the nucleic acid content of PRRSV-1 was extremely low (≤10^1^ copies/μL), the detection rate was lower than that of commercially available kits (Table 5). However, when the nucleic acid concentration is ≥10^2^ copies/μL, the detection rate of PRRSV-1 is basically consistent with the commercially available kit, with a conformity rate of 97.9–100% (Table 5). For PRRSV-2, HP-PRRSV, and NL-PRRSV, even at a viral nucleic acid concentration of 10^1^ copies/μL, the agreement rate between this method and the detection results of commercially available kits is 93.8–95.8% (Table 5). In terms of specificity, our method is basically consistent with the detection results of the kit, with a conformity rate of 95.8–100% (Supplementary Table S6). In addition, in terms of effectiveness, the consistency rate between this differential diagnostic method and the kit is 93.3–100% (Table 6). In summary, the duplex qPCR method established by our research institute for differential diagnosis of PRRSV has high consistency with the detection results of commercially available kits.

**Table 5:** The consistency test results of sensitivity between this method and commercially available reagent kits.

Template	Plasmid concentration(copies/μL)	VetMAX PRRSV EU&NA 2.0 Kit			
			Positive	Negative	Agreement rate
PRRSV-1 (PMD-EU)	10^1^	Positive	2	0	54.2%
		Negative	22	24	
	10^2^	Positive	23	0	97.9%
		Negative	1	24	
	10^3^	Positive	24	0	100.0%
		Negative	0	24	
PRRSV-2 (PMD-US)	10^1^	Positive	21	0	93.8%
		Negative	3	24	
	10^2^	Positive	23	0	97.9%
		Negative	1	24	
	10^3^	Positive	24	0	100.0%
		Negative	0	24	
HP-PRRSV (PMD-HP)	10^1^	Positive	22	0	95.8%
		Negative	2	24	
	10^2^	Positive	22	0	95.8%
		Negative	2	24	
	10^3^	Positive	24	0	100.0%
		Negative	0	24	
NL-PRRSV (PMD-NL)	10^1^	Positive	21	0	93.8%
		Negative	3	24	
	10^2^	Positive	23	0	97.9%
		Negative	1	24	
	10^3^	Positive	24	0	100.0%
		Negative	0	24	

**Table 6:** The consistency test results of effectiveness between this method and commercially available reagent kits.

Template	VetMAX PRRSV EU&NA 2.0 Kit			
		Positive	Negative	Agreement rate
PRRSV-1	Positive	13	0	93.3%
	Negative	2	15	
PRRSV-2	Positive	14	0	96.7%
	Negative	1	15	
HP-PRRSV	Positive	15	0	100.0%
	Negative	0	15	
NL-PRRSV	Positive	14	0	96.7%
	Negative	1	15	

### 3.7 Clinical sample detection

Three hundred and sixteen samples collected from different swine farms in Guangdong Province during 2021–2023 were tested using the method established in this study, including serum, blood, and clinical tissue samples (Table 7). Among the 316 samples, for blood samples, the PRRSV-2 positive rate was 11.54% (9/78), and all positive samples belonged to HP-PRRSV (Table 7). For tissue samples, the positive rate for PRRSV-2 was 45.32% (63/139), with HP-PRRSV and NL-PRRSV being 28.78% (40/139) and 2.16% (3/139), respectively (Table 7). On the contrary, positive samples were not detected in all serum samples.

**Table 7:** Detection results of different PPRSV in different types of pig samples.

Samples type	Number of positive samples/Number of total samples (positive rate %, 95 CI)			
	PRRSV-1	PRRSV-2	HP-PRRSV	NL-PRRSV
Blood	0/78 (0%, 0–4.6)	9/78 (11.54%, 5.4–20.8)	9/78 (11.54%, 5.4–20.8)	0/78 (0%, 0–4.6)
Serum	0/99 (0%, 0–3.7)	0/99 (0%, 0–3.7)	0/99 (0%, 0–3.7)	0/99 (0%, 0–3.7)
Tissue	0/139 (0%, 0–2.6)	63/139 (45.32%, 36.9–54.0)	40/139 (28.78%, 21.4–37.1)	3/139 (2.16%, 0.4–6.2)
Total	0/316 (0%, 0–1.2)	72/316 (22.78%, 18.3–27.8)	49/316 (15.51%, 11.7–20)	3/316 (0.95%, 0.2–2.7)

In addition, the PRRSV-1 positive rate was 0% (0/316), and the PRRSV-2 positive rate was 22.78% (72/316) (Table 8). Among all surveyed areas, the prevalence of PRRSV-2 in Yunfu was the highest, reaching 78.43% (40/51), followed by Heyuan (16.25%, 13/80) (Table 8). It is worth noting that our results also indicated a positive rate of 15.51% (49/316) for highly pathogenic PRRSV, while 0.95% (3/316) for NADC30-like PRRSV (Table 8). Moreover, Yunfu is also the city with the most severe HP-PRRSV epidemic, followed by Shanwei (Table 8). However, during this investigation, no co-infection of different strains was found.

**Table 8:** Summary of detection of different types of PRRSV in 316 pig samples.

City	Number of positive samples/Number of total samples (positive rate %, 95 CI)			
	PRRSV-1	PRRSV-2	HP-PRRSV	NL-PRRSV
Chaozhou	0/3 (0%, 0–70.8)	0/3 (0%, 0–70.8)	0/3 (0%, 0–70.8)	0/3 (0%, 0–70.8)
Foshan	0/2 (0%, 0–84.2)	0/2 (0%, 0–84.2)	0/2 (0%, 0–84.2)	0/2 (0%, 0–84.2)
Guangzhou	0/1 (0%, 0–97.5)	0/1 (0%, 0–97.5)	0/1 (0%, 0–97.5)	0/1 (0%, 0–97.5)
Heyuan	0/80 (0%, 0–4.5)	13/80 (16.25%, 8.9–26.2)	0/80 (0%, 0–4.5)	0/80 (0%, 0–4.5)
Jiangmen	0/49 (0%, 0–7.3)	1/49 (2.04%, 0.1–10.9)	0/49 (0%, 0–7.3)	1/49 (2.04%, 0.1–10.9)
Maoming	0/13 (0%, 0–24.7)	8/13 (61.54%, 31.6–86.1)	0/13 (0%, 0–24.7)	2/13 (15.38%, 1.9–45.4)
Meizhou	0/5 (0%, 0–52.2)	0/5 (0%, 0–52.2)	0/5 (0%, 0–52.2)	0/5 (0%, 0–52.2)
Qingyuan	0/25 (0%, 0–13.7)	0/25 (0%, 0–13.7)	0/25 (0%, 0–13.7)	0/25 (0%, 0–13.7)
Shantou	0/4 (0%, 0–60.2)	0/4 (0%, 0–60.2)	0/4 (0%, 0–60.2)	0/4 (0%, 0–60.2)
Shanwei	0/20 (0%, 0–16.8)	9/20 (45%, 23.1–68.5)	9/20 (45%, 23.1–68.5)	0/20 (0%, 0–16.8)
Shaoguan	0/2 (0%, 0–84.2)	0/2 (0%, 0–84.2)	0/2 (0%, 0–84.2)	0/2 (0%, 0–84.2)
Yangjiang	0/59 (0%, 0–6.1)	1/59 (1.69%, 0–9.1)	0/59 (0%, 0–6.1)	0/59 (0%, 0–6.1)
Yunfu	0/51 (0%, 0–7.0)	40/51 (78.43%, 64.7–88.7)	40/51 (78.43%, 64.7–88.7)	0/51 (0%, 0–7.0)
Zhaoqing	0/2 (0%, 0–84.2)	0/2 (0%, 0–84.2)	0/2 (0%, 0–84.2)	0/2 (0%, 0–84.2)
Total	0316 (0%, 0–1.2)	72/316(22.78%, 18.3–27.8)	49/316(15.51%, 11.7–20)	3/316 (0.95%, 0.2–2.7)

### 3.8 PRRSV genetic characteristics in Guangdong

To further investigate the genetic characteristics of PRRSV in the surveyed regions of Guangdong Province, representative samples (*n* = 13) from each region were selected for amplification and sequencing of the complete ORF5 fragment. A total of 13 complete ORF5 gene sequences of PRRSV were successfully obtained, each of which was 603 nt in size, and have been archived in the GenBank database, with accession numbers OR539223 through OR539235.

**Figure 5:**
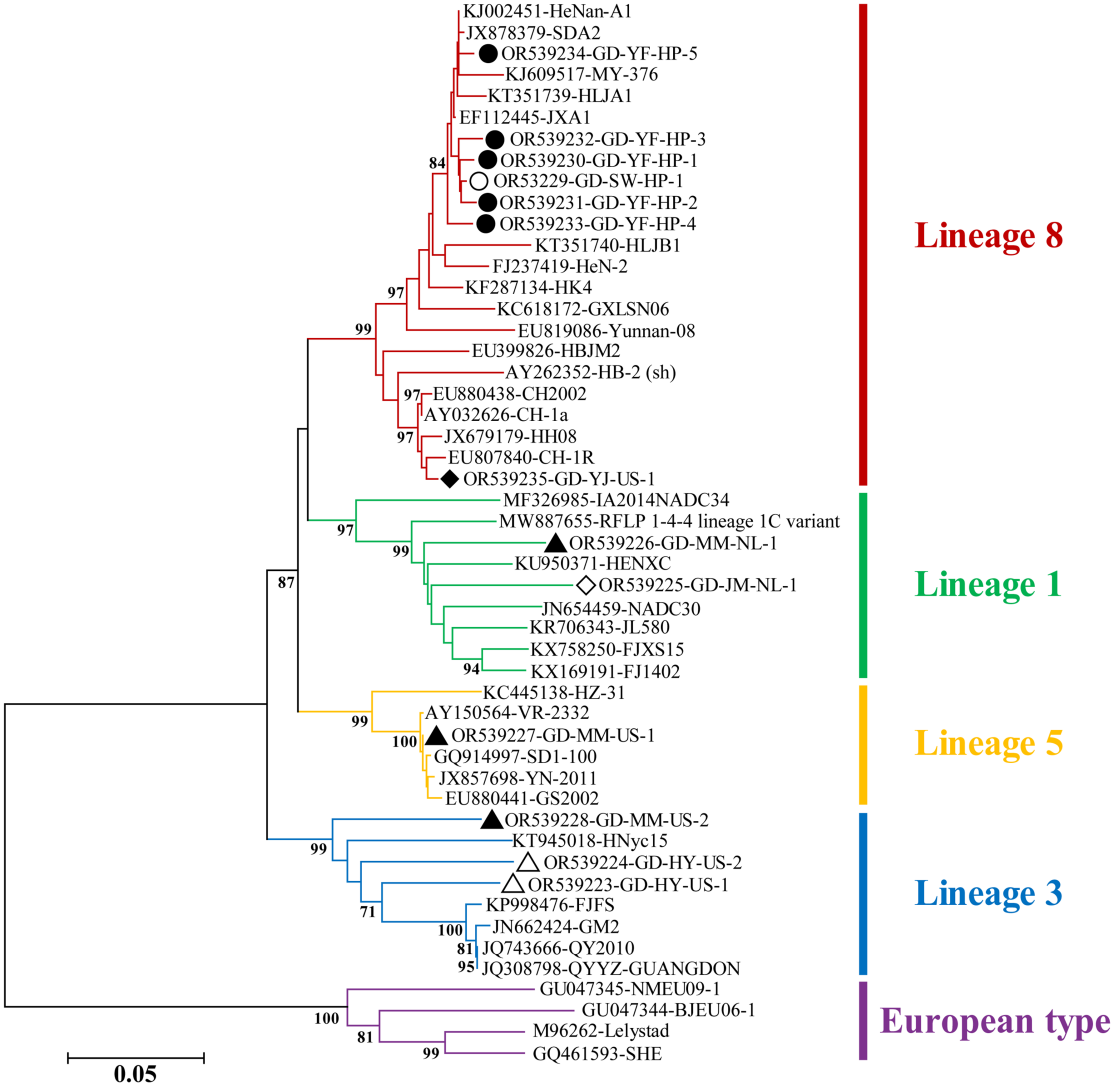
Phylogenetic tree based on ORF5 gene of PRRSV. The NJ tree was constructed using the Maximum Composite Likelihood model and bootstrapped at 1000 replicates. And bootstrap values less than 70 are not displayed. The different genotypes or lineages are represented by different colors as indicated in the figures. The PRRSV strains from Yunfu and Shanwei are indicated by black solid and hollow circles, respectively. The PRRSV strains from Yangjiang and Jiangmen are indicated by black solid and hollow rhombuses, respectively. The PRRSV strains from Maoming and Heyuan are indicated by black solid and hollow triangles, respectively.

Phylogenetic analysis revealed that the strains collected from Guangdong belonged to four lineages (1, 3, 5, and 8), with lineage 8 being the dominant strain, particularly sub-lineage 8.3 (Figure 5). Further analysis showed that highly pathogenic PRRSV was mainly prevalent in the Yunfu and Shanwei regions, while classical PRRSV was predominantly observed in the Yangjiang region. Additionally, lineage 3 strains were predominant in the Heyuan region. Notably, PRRSV circulating in the pig population in Maoming exhibited high genetic diversity, belonging to three different lineages: 1, 3, and 5 (Figure 5).

Nucleotide sequence analysis revealed that the HP-PRRSV strains shared 98.7–99.3% complete nucleotide identity with highly pathogenic strain JXA1. However, the classical strain from this study shared 99.2% nucleotide identity with strain CH-1a. In addition, the strains of lineage 1, 3 and 5 shared 90.2–91.7%, 90.9–92.9 and 99.8% nucleotide identity with the reference strains of same lineage, respectively.

## 4 Discussion

In the past 20 years, PRRSV has remained one of the most significant diseases affecting the Chinese pig industry. Due to its diverse modes of transmission, frequent genetic mutations and recombination, immune suppression and evasion, antibody-dependent enhancement effect, and the lack of effective vaccines and antiviral drugs, substantial breakthroughs in PRRSV eradication in China have yet to be achieved. Currently, PRRSV prevention and control mainly rely on immunization with attenuated vaccines (17), which exhibit limited cross-protection against heterologous strains.

To guide the formulation of vaccine immunization strategies, this study developed two duplex-fluorescence quantitative PCR methods based on SYBR Green I, capable of simultaneously detecting and distinguishing PRRSV-1 and PRRSV-2, as well as HP-PRRSV and NL-PRRSV. The method demonstrated a lower detection limit of 10^1^ copies/μL to 10^2^ copies/μL, along with good specificity and reproducibility. Importantly, this cost-effective method does not require stringent experimental conditions and can be easily implemented in primary pig farms, enabling the formulation of personalized PRRSV immunization strategies and aiding in the prevention and control of PRRSV.

Previous studies have indicated that the PRRSV nucleic acid positivity rate in certain regions of northern China (Shandong, Henan) (7, 18–21) (18.0–36.7%) was higher than that in southern China (Guangdong) (12.07%) (22–24). In contrast, in this study, the PRRSV positivity rate in certain regions of Guangdong Province was 22.78%, which is similar to the positivity rate in northern regions of China. This discrepancy may be attributed to variations in sample sources, quantities, and detection methods.

Further phylogenetic analysis showed that, similar to the findings of Zhang et al. (25), PRRSV lineages 1, 3, 5, and 8 were prevalent in pig farms in certain regions of Guangdong Province in 2022, with lineage 8 strains being the dominant ones. In this study, the strain of lineage 8 was mainly prevalent in western Guangdong, primarily concentrated in Yunfu, while only sporadically present in eastern Guangdong. This finding is consistent with the investigation results of PRRSV in Guangdong in recent years (23, 26–28). It is worth noting that the genetic diversity of PRRSV in Maoming is the most abundant, involving lineages 1, 3, and 5.

However, it is noteworthy that we did not observe co-infection of different PRRSV lineages in the investigated pig farms in certain regions of Guangdong Province. This suggests that the occurrence of mixed infections with multiple PRRSV lineages in the pig population of the surveyed regions in Guangdong Province is rare.

In general, despite the availability of commercial PRRSV vaccines, PRRS remains a significant infectious disease in Chinese swine herds due to its complex genetic background and weak cross-protection among different lineages. Therefore, regular genetic lineage surveys of PRRSV in the areas surrounding pig farms are necessary to prevent and control disease outbreaks. The duplex real-time PCR detection method developed in this study for PRRSV-1 and PRRSV-2, as well as HP-PRRSV and NL-PRRSV, exhibits good sensitivity, specificity, and reproducibility. Although this method still has some minor drawbacks compared to currently commercialized detection kits, it remains a fast, convenient, and cost-effective clinical tool for effectively identifying PRRSV genetic typing near agricultural facilities. This is of great significance for developing effective PRRSV immunization strategies in clinical settings.

Additionally, due to the rich genetic diversity of PRRSV, such as the further division of highly pathogenic PRRSV into different lineages (26, 29), there is a need to consider how to more finely distinguish the lineages of strains. Furthermore, further research is needed to determine whether this method is applicable for identifying global PRRSV strains or novel recombinant strains. In future work, this differential diagnosis method will be further optimized.

## 5 Conclusion

In conclusion, the duplex real-time qPCR method developed in this research for the detection of PRRSV-1 and PRRSV-2, along with HP-PRRSV and NL-PRRSV, demonstrates excellent sensitivity, specificity, and reproducibility. This method offers a swift, user-friendly, and economical clinical solution for the identification of PRRSV genetic lineages in areas surrounding pig farms, thereby facilitating the implementation of targeted immunization strategies and improving PRRSV control and prevention efforts.

## Data Availability

The datasets presented in the study are deposited in the GenBank repository. The names in the repository/repositories and accession number(s) can be found in the article/Supplementary material.
