# Supplementary material for: Development of SYBR green I-based real-time qPCR differential diagnosis assays for porcine reproductive and respiratory syndrome virus typing in Guangdong province
Source: Front Vet Sci. 2025 Mar 5;12:1495128. doi: 10.3389/fvets.2025.1495128 (PMC11921047; doi:10.3389/fvets.2025.1495128)
Supplement: Supplementary file 1 [file Table_1.docx]

Supplementary Material

# Supplementary Tables

**Supplementary Table S1. The information of reference sequences used for primer design**

| Strain name | Virus genotype | Lineage | GenBank Accession no. | Country | Collection year |
| --- | --- | --- | --- | --- | --- |
| SHE | I | no | GQ461593 | China | 2008 |
| GZ11-G1 | I | no | KF001144 | China | 2011 |
| NVDC-NM1-2011 | I | no | JX187609 | China | 2011 |
| BJEU06-1 | I | no | GU047344 | China | 2006 |
| FJEU13 | I | no | KP860912 | China | 2015 |
| NVDC-NM3 | I | no | KC492505 | China | 2011 |
| NVDC-FJ | I | no | KC492506 | China | 2011 |
| NVDC-NM2 | I | no | KC492504 | China | 2011 |
| NMEU09-1 | I | no | GU047345 | China | 2009 |
| FJQEU14 | I | no | KP860913 | China | 2015 |
| HLJB1 | I | no | KT351740 | China | 2013 |
| PRRSV2/CN/F1004/2017 | II | 3 | MT416544 | China | 2017 |
| HLJA1 | II | 8 | KT351739 | China | 2013 |
| Henan-A12 | II | 8 | KJ819934 | China | 2014 |
| MY-486 | II | 8 | KJ609516 | China | 2013 |
| Henan-A14 | II | 8 | KJ819936 | China | 2014 |
| HeNan-A1 | II | 8 | KJ002451 | China | 2013 |
| SDA2 | II | 8 | JX878379 | China | 2011 |
| SDA3 | II | 8 | JX878380 | China | 2011 |
| JXA1 | II | 8 | EF112445 | China | 2006 |
| SHH | II | 8 | EU106888 | China | 2006 |
| NVDC-CQ4-2012 | II | 8 | KP771777 | China | 2012 |
| CWZ-1-F3 | II | 8 | FJ889130 | China | 2008 |
| HUN4 | II | 8 | EF635006 | China | 2006 |
| FZ06A | II | 8 | MF370557 | China | 2006 |
| GDBY1 | II | 8 | GQ374442 | China | 2008 |
| GD-HD | II | 8 | KP793736 | China | 2011 |
| Jsyx | II | 8 | EU939312 | China | 2006 |
| JXwn06-81c | II | 8 | HQ233604 | China | 2006 |
| JXwn06 | II | 8 | EF641008 | China | 2006 |
| TJ | II | 8 | EU860248 | China | 2006 |
| JXM40 | II | 8 | GQ499194 | China | 2008 |
| JXM20 | II | 8 | GQ499193 | China | 2008 |
| JXM80 | II | 8 | GQ499196 | China | 2008 |
| JXM60 | II | 8 | GQ499195 | China | 2008 |
| JXM100 | II | 8 | GQ475526 | China | 2008 |
| HUB1 | II | 8 | EF075945 | China | 2006 |
| HUB2 | II | 8 | EF112446 | China | 2006 |
| GD | II | 8 | EU109503 | China | 2006 |
| BJSY-1 | II | 8 | FJ950744 | China | 2007 |
| BJSY07 | II | 8 | HM011104 | China | 2007 |
| BJBLZ | II | 8 | FJ950745 | China | 2007 |
| NVDC-CQ3-2012 | II | 8 | KP771775 | China | 2012 |
| HEB1 | II | 8 | EF112447 | China | 2006 |
| Jiangxi-3 | II | 8 | EU200961 | China | 2006 |
| CBB-1-F3 | II | 8 | FJ889129 | China | 2008 |
| NVDC-CQ1-2012 | II | 8 | KP771747 | China | 2012 |
| BJsy06 | II | 8 | EU097707 | China | 2006 |
| NX06 | II | 8 | EU097706 | China | 2006 |
| HLM-09 | II | 8 | HQ843179 | China | 2009 |
| SD-09 | II | 8 | HQ843180 | China | 2009 |
| GDQY1VP100 | II | 8 | JN387274 | China | 2011 |
| GDQY1VP80 | II | 8 | JN387273 | China | 2010 |
| GDQY1VP65 | II | 8 | JN387272 | China | 2010 |
| TP | II | 8 | EU864233 | China | 2006 |
| GDQY1 | II | 8 | JN387271 | China | 2007 |
| TP P90 | II | 8 | GU232737 | China | 2008 |
| TP P60 | II | 8 | GU232736 | China | 2008 |
| YN9 | II | 8 | GU232738 | China | 2008 |
| GD | II | 8 | EU825724 | China | 2007 |
| GD isolation-source 100th passage | II | 8 | GU143913 | China | 2009 |
| 07BJ | II | 8 | FJ393459 | China | 2007 |
| NVDC-CQ2-2012 | II | 8 | KP771776 | China | 2012 |
| HV | II | 8 | JX317648 | China | 2007 |
| HN-HW | II | 8 | FJ797690 | China | 2006 |
| NM1 | II | 8 | EU860249 | China | 2007 |
| 07NM | II | 8 | FJ393456 | China | 2007 |
| 07HEBTJ | II | 8 | FJ393458 | China | 2007 |
| 07HEN | II | 8 | FJ393457 | China | 2007 |
| BJSD | II | 8 | FJ950747 | China | 2007 |
| Henan-1 | II | 8 | EU200962 | China | 2007 |
| SX-09 | II | 8 | HQ843181 | China | 2009 |
| HLJ-09 | II | 8 | HQ843178 | China | 2009 |
| 08HuN | II | 8 | GU169411 | China | 2008 |
| LN | II | 8 | EU109502 | China | 2006 |
| TJbd14-1 | II | 8 | KP742986 | China | 2014 |
| TJbd14-2 | II | 8 | KP742987 | China | 2014 |
| NVDC-R224-2014 | II | 8 | KP771783 | China | 2014 |
| GDHY | II | 8 | KY488476 | China | 2015 |
| XJu-1 | II | 8 | KF815525 | China | 2012 |
| HB18-36 | II | 8 | MW627193 | China | 2018 |
| HB19-12 | II | 8 | MW651975 | China | 2019 |
| BJ | II | 8 | EU825723 | China | 2007 |
| NVDC-BJ7-2012 | II | 8 | KP771758 | China | 2012 |
| NVDC-BJ8-2012 | II | 8 | KP771757 | China | 2012 |
| NVDC-CQ-2008 | II | 8 | KP771755 | China | 2008 |
| 11FS12-GD | II | 8 | JX215554 | China | 2011 |
| 11FS11-GD | II | 8 | JX215551 | China | 2011 |
| 11GZ-GD | II | 8 | JX235370 | China | 2011 |
| 10GZ-GD | II | 8 | JX192633 | China | 2010 |
| XH-GD | II | 8 | EU624117 | China | 2007 |
| WUH3 | II | 8 | HM853673 | China | 2008 |
| SC/NJ 2016 | II | 8 | MF818049 | China | 2016 |
| NVDC-HeB-2008 | II | 8 | KP771754 | China | 2008 |
| AH0701 | II | 8 | GU461292 | China | 2007 |
| SD16 | II | 8 | JX087437 | China | 2012 |
| FZ16A | II | 8 | KY761966 | China | 2016 |
| NVDC-BJ4-2012 | II | 8 | KP771761 | China | 2012 |
| NVDC-BJ6-2012 | II | 8 | KP771759 | China | 2012 |
| NVDC-BJ5-2012 | II | 8 | KP771760 | China | 2012 |
| NVDC-13SXJC-2014 | II | 8 | KP771780 | China | 2014 |
| NVDC-SXJC-2013 | II | 8 | KP771740 | China | 2013 |
| HNxa14 | II | 8 | KT022071 | China | 2014 |
| HEB 20130008-14 | II | 8 | KP771752 | China | 2013 |
| JXA1 P45 | II | 8 | FJ548851 | China | 2006 |
| JXA1 P15 | II | 8 | FJ548855 | China | 2006 |
| JXA1 P10 | II | 8 | FJ548854 | China | 2006 |
| JXA1 P70 | II | 8 | FJ548852 | China | 2006 |
| JXA1 P80 | II | 8 | FJ548853 | China | 2006 |
| JXA1-P100 | II | 8 | KC422725 | China | 2009 |
| JXA1-P160 | II | 8 | KC422731 | China | 2009 |
| JXA1-P150 | II | 8 | KC422730 | China | 2009 |
| JXA1-P140 | II | 8 | KC422729 | China | 2009 |
| JXA1-P170 | II | 8 | JQ804986 | China | 2009 |
| JXA1-P130 | II | 8 | KC422728 | China | 2009 |
| JXA1-P110 | II | 8 | KC422726 | China | 2009 |
| JXA1-P120 | II | 8 | KC422727 | China | 2009 |
| HENPDS-2 | II | 8 | KU950370 | China | 2015 |
| GX1003 | II | 8 | JX912249 | China | 2010 |
| HNHK2 | II | 8 | KY488474 | China | 2014 |
| GDST | II | 8 | KY498542 | China | 2014 |
| GDZQ | II | 8 | KY488473 | China | 2014 |
| HNHK1 | II | 8 | KY488475 | China | 2014 |
| NVDC-SD1-2014 | II | 8 | KP771738 | China | 2014 |
| NT2 | II | 8 | KP179403 | China | 2012 |
| NT1 | II | 8 | KP179402 | China | 2012 |
| Shaanxi-2 | II | 8 | HQ401282 | China | 2007 |
| NT3 | II | 8 | KP179404 | China | 2012 |
| FJZH | II | 8 | KP998478 | China | no |
| GX1002 | II | 8 | JQ955658 | China | 2010 |
| WUH4 | II | 8 | JQ326271 | China | 2011 |
| 11XX-GD | II | 8 | JX235367 | China | 2011 |
| 11NZ-GD | II | 8 | JX217036 | China | 2011 |
| NVDC-BJ9-2012 | II | 8 | KP771756 | China | 2012 |
| NVDC-HuNCS-2014 | II | 8 | KP771781 | China | 2014 |
| NVDC-SD2-2012 | II | 8 | KP771768 | China | 2012 |
| NVDC-shh01-2014 | II | 8 | KP771736 | China | 2014 |
| JL-04/12 | II | 8 | JX177644 | China | 2012 |
| NVDC-HeB1-2011 | II | 8 | KP771749 | China | 2011 |
| 11SH1-GD | II | 8 | JX235366 | China | 2011 |
| HENZK-1 | II | 8 | KU950373 | China | 2014 |
| FJCH | II | 8 | KP998477 | China | no |
| HNyc13 | II | 8 | KT022072 | China | 2013 |
| NVDC-BJ3-2012 | II | 8 | KP771762 | China | 2012 |
| HB19-18 | II | 8 | MW651976 | China | 2019 |
| YD | II | 8 | JF748717 | China | 2009 |
| GDMM | II | 8 | KY488472 | China | 2014 |
| HB2014001 | II | 8 | KM261784 | China | 2014 |
| NVDC-MD1-2013 | II | 8 | KP771751 | China | 2013 |
| NVDC-BJPG-2013 | II | 8 | KP771743 | China | 2013 |
| NVDC-SDXX-2013 | II | 8 | KP771741 | China | 2013 |
| 11SH-GD | II | 8 | JX235365 | China | 2011 |
| GDQJ | II | 8 | GQ374441 | China | 2007 |
| 07QN | II | 8 | FJ394029 | China | 2007 |
| SD-CXA/2008 | II | 8 | GQ359108 | China | 2008 |
| 10-10QN | II | 8 | JQ663556 | China | 2010 |
| NVDC-HBCZ-2013 | II | 8 | KP771742 | China | 2013 |
| SX-1 | II | 8 | GQ857656 | China | 2009 |
| 09SD | II | 8 | JF268678 | China | 2009 |
| 09HEB | II | 8 | JF268679 | China | 2009 |
| 09SC | II | 8 | JF268672 | China | 2009 |
| SX2009 | II | 8 | FJ895329 | China | 2009 |
| 09HUB2 | II | 8 | JF268683 | China | 2009 |
| 09HUB1 | II | 8 | JF268682 | China | 2009 |
| ZP-1 | II | 8 | HM016159 | China | 2009 |
| HuN | II | 8 | EF517962 | China | no |
| NVDC-NM-2008 | II | 8 | KP771779 | China | 2008 |
| GDQY2 | II | 8 | GU454850 | China | 2007 |
| CG | II | 8 | EU864231 | China | 2007 |
| HLJHL | II | 8 | HM189676 | China | 2009 |
| 09DB2 | II | 8 | JF268681 | China | 2009 |
| 09DB1 | II | 8 | JF268677 | China | 2009 |
| 10-10JL | II | 8 | JQ663554 | China | 2012 |
| NVDC-JS2-2011 | II | 8 | JQ715698 | China | 2011 |
| GDQY | II | 8 | KY488478 | China | 2015 |
| HN2007 | II | 8 | EU880437 | China | 2007 |
| XL2008 | II | 8 | EU880436 | China | 2008 |
| GS2008 | II | 8 | EU880431 | China | 2008 |
| YN2008 | II | 8 | EU880435 | China | 2008 |
| SX2007 | II | 8 | EU880434 | China | 2007 |
| PRRSV2/CN/101806/2018 | II | 8 | MT416545 | China | 2018 |
| HUN-2014 | II | 8 | KP330232 | China | 2014 |
| BJPG | II | 8 | FJ950746 | China | 2007 |
| GDGZ | II | 8 | KY488471 | China | 2014 |
| GDJM | II | 8 | KY488470 | China | 2014 |
| NVDC-SHH02-2014 | II | 8 | KP771735 | China | 2014 |
| GX09-32 | II | 8 | HM214915 | China | no |
| 09HUN2 | II | 8 | JF268674 | China | 2009 |
| HNP5 | II | 8 | KT445876 | China | 2014 |
| HN07-1 | II | 8 | KX766378 | China | 2007 |
| WUH1 | II | 8 | EU187484 | China | no |
| GX1001 | II | 8 | JQ955657 | China | 2011 |
| HEB 20130008-13 | II | 8 | KP771753 | China | 2013 |
| SCwhn09CD | II | 8 | JN836553 | China | 2009 |
| NVDC-HeB1-2013 | II | 8 | KP771745 | China | 2013 |
| NVDC-HeB2-2013 | II | 8 | KP771744 | China | 2013 |
| NVDC-SC1-2014 | II | 8 | KP771739 | China | 2014 |
| NVDC-R38-2014 | II | 8 | KP771782 | China | 2014 |
| NVDC-SD1-2012 | II | 8 | KP771769 | China | 2012 |
| JXja15 | II | 8 | KR149645 | China | no |
| 10-10GX-3 | II | 8 | JQ663560 | China | 2010 |
| GX10-42 | II | 8 | JQ309822 | China | no |
| 10SS-GD | II | 8 | JX192638 | China | 2010 |
| 10HD-GD | II | 8 | JX215553 | China | 2010 |
| 10FS1-GD | II | 8 | JX192635 | China | 2010 |
| 10-10HEB-3 | II | 8 | JQ663553 | China | 2010 |
| 09HEN1 | II | 8 | JF268684 | China | 2009 |
| 10-10GX-2 | II | 8 | JQ663559 | China | 2010 |
| 10-10GX-4 | II | 8 | JQ663561 | China | 2010 |
| 10-10HEB-2 | II | 8 | JQ663552 | China | 2010 |
| JN-HS | II | 8 | HM016158 | China | 2008 |
| 09HEN2 | II | 8 | JF268680 | China | 2009 |
| 09JS | II | 8 | JF268675 | China | 2009 |
| 10-LW2-6 | II | 8 | JQ663563 | China | 2010 |
| 10-LW1-13 | II | 8 | JQ663557 | China | 2010 |
| 10-LW8-1 | II | 8 | JQ663568 | China | 2010 |
| 10-LW7-1 | II | 8 | JQ663567 | China | 2010 |
| 10-LW6-6 | II | 8 | JQ663566 | China | 2010 |
| 10-10LW5-1 | II | 8 | JQ663565 | China | 2010 |
| 10-LW3-7 | II | 8 | JQ663564 | China | 2010 |
| 10-10HEB-1 | II | 8 | JQ663551 | China | 2010 |
| 10-10FUJ-1 | II | 8 | JQ663546 | China | 2010 |
| 10-10GX-5 | II | 8 | JQ663562 | China | 2010 |
| NVDC-HeB2-2011 | II | 8 | KP771765 | China | 2011 |
| 10-10BJ-2 | II | 8 | JQ663543 | China | 2010 |
| GX10-48 | II | 8 | JQ309823 | China | no |
| 10-10BJ-4 | II | 8 | JQ663544 | China | 2010 |
| 10-10GX-1 | II | 8 | JQ663558 | China | 2010 |
| 10-10BJ-1 | II | 8 | JQ663541 | China | 2010 |
| 10-10SD | II | 8 | JQ663555 | China | 2010 |
| 10-10BJ-5 | II | 8 | JQ663545 | China | 2010 |
| NVDC-YN-2011 | II | 8 | KP771767 | China | 2011 |
| NVDC-HuN-2011 | II | 8 | KP771770 | China | 2012 |
| 10-10FUJ-5 | II | 8 | JQ663550 | China | 2010 |
| 10QY-GD | II | 8 | JX215552 | China | 2010 |
| 10SJ-GD | II | 8 | JX192637 | China | 2010 |
| 10-10JX | II | 8 | JQ663540 | China | 2010 |
| 10-10FUJ-4 | II | 8 | JQ663549 | China | 2010 |
| 10-10FUJ-3 | II | 8 | JQ663548 | China | 2010 |
| 10-10FUJ-2 | II | 8 | JQ663547 | China | 2010 |
| FS | II | 8 | JF796180 | China | 2010 |
| NVDC-BJ1-2012 | II | 8 | KP771764 | China | 2012 |
| NVDC-BJ2-2012 | II | 8 | KP771763 | China | 2012 |
| 10ZQ-GD | II | 8 | JX192639 | China | 2010 |
| DC | II | 8 | JF748718 | China | 2010 |
| JX | II | 8 | JX317649 | China | 2010 |
| NVDC-HeB2-2012 | II | 8 | KP771772 | China | 2012 |
| Shanxi-6 | II | 8 | KJ855518 | China | 2010 |
| 10BY-GD | II | 8 | JX192636 | China | 2010 |
| 10FS-GD | II | 8 | JX192634 | China | 2010 |
| NVDC-GD-2011 | II | 8 | KP771766 | China | 2011 |
| NVDC-BJ1-2011 | II | 8 | KP771778 | China | 2011 |
| NVDC-HeN-2012 | II | 8 | KP771771 | China | 2012 |
| NVDC-CQ1-2011 | II | 8 | KP771746 | China | 2011 |
| NVDC-CQ3-2011 | II | 8 | KP771774 | China | 2011 |
| 09BJ | II | 8 | JF268676 | China | 2009 |
| FJOU | II | 8 | KP998479 | China | no |
| GX09-29 | II | 8 | HM214914 | China | no |
| FJYR | II | 8 | KT804696 | China | no |
| GDSG | II | 8 | KY488479 | China | 2015 |
| Henan-A8 | II | 8 | KJ534543 | China | 2013 |
| Henan-A7 | II | 8 | KJ534542 | China | 2013 |
| 09HUN1 | II | 8 | JF268673 | China | 2009 |
| HENZZ-8 | II | 8 | KU950375 | China | 2015 |
| QTX | II | 8 | KX357708 | China | 2016 |
| WUH2 | II | 8 | EU678352 | China | no |
| GDHZ | II | 8 | KY488477 | China | 2015 |
| HB18-41 | II | 8 | MT268280 | China | 2018 |
| GXYL1403 | II | 8 | MN660069 | China | 2014 |
| Henan-A6 | II | 8 | KJ534541 | China | 2013 |
| Henan-A5 | II | 8 | KJ534540 | China | 2013 |
| GX09-16 | II | 8 | HM214913 | China | no |
| NMG2014 | II | 8 | KM000066 | China | 2014 |
| Henan-A13 | II | 8 | KJ819935 | China | 2014 |
| NVDC-SD4-2014 | II | 8 | KP771784 | China | 2014 |
| NJ-1106 | II | 8 | JX880029 | China | 2011 |
| NT0801 | II | 8 | HQ315836 | China | 2008 |
| NT0801 P10 | II | 8 | KJ523894 | China | 2008 |
| NT0801 P30 | II | 8 | KJ523895 | China | 2009 |
| NT0801 P80 | II | 8 | KJ523897 | China | 2011 |
| NT0801 P50 | II | 8 | KJ523896 | China | 2010 |
| BJ0706 | II | 8 | GQ351601 | China | 2007 |
| NB/04 | II | 8 | FJ536165 | China | 2004 |
| HB-1/3.9c | II | 8 | HQ233605 | China | 2002 |
| HB-1/3.9 | II | 8 | EU360130 | China | 2002 |
| GD3 | II | 8 | GU269541 | China | 2005 |
| SHB | II | 8 | EU864232 | China | 2005 |
| NVDC-HeB1-2012 | II | 8 | KP771773 | China | 2012 |
| NVDC-SD6-2014 | II | 8 | KP771737 | China | 2014 |
| SCwhn14DY | II | 8 | KT819203 | China | 2014 |
| 17-ZJ-HZ | II | 8 | MF770574 | China | 2017 |
| 10HN-GD | II | 8 | JX192632 | China | 2010 |
| HeNan-A2 | II | 8 | KJ002452 | China | 2013 |
| SDZZ | II | 8 | KY373217 | China | 2014 |
| MY-376 | II | 8 | KJ609517 | China | 2013 |
| HeNan-A9 | II | 8 | KJ546412 | China | 2013 |
| AHBZ | II | 8 | KY373216 | China | 2014 |
| NVDC-BJ2-2011 | II | 8 | KP771748 | China | 2011 |
| GXNN1396-P96 | II | 8 | MN660068 | China | 2013 |
| GXNN1396-p3 | II | 8 | MN660067 | China | 2013 |
| GDDX-2018 | II | 8 | MT379661 | China | 2018 |
| PRRSV2/CN/GDDX/2018 | II | 8 | MT721741 | China | 2018 |
| PRRSV2/CN/X2984/2018 | II | 8 | MT416541 | China | 2018 |
| PRRSV2/CN/N9185/2018 | II | 8 | MT416542 | China | 2018 |
| PRRSV2/CN/F1228/2017 | II | 8 | MT416543 | China | 2017 |
| XF1129 | II | 8 | KT180169 | China | 2013 |
| GZgy15-1 | II | 8 | KT358728 | China | 2015 |
| HiNZWQ | II | 8 | KY373215 | China | 2014 |
| GSWW/CHA 2015 | II | 8 | KX767091 | China | 2015 |
| NVDC-GD2-2011 | II | 8 | JQ715697 | China | 2011 |
| FJNP2017 | II | 8 | MH046842 | China | 2017 |
| GDZS2016 | II | 8 | MH046843 | China | 2016 |
| 15LY01-FJ | II | 8 | KU215416 | China | no |
| 14LY02-FJ | II | 8 | KP780882 | China | 2014 |
| HENXX-9 | II | 8 | KY290748 | China | 2016 |
| TJnh1501 | II | 8 | KX510269 | China | no |
| 15LY02-FJ | II | 8 | KU215417 | China | no |
| 14LY01-FJ | II | 8 | KP780881 | China | 2014 |
| SD-YL1712 | II | 8 | MT708500 | China | 2017 |
| FJE1 | II | 8 | KP998475 | China | no |
| FJXS15 | II | 1 | KX758250 | China | 2015 |
| FJW05 | II | 1 | KP860911 | China | 2013 |
| SCya17 | II | 3 | MH324400 | China | 2017 |
| GDYDZZZ | II | 3 | KY745901 | China | 2016 |
| ZJXS1412 | II | 8 | MF669722 | China | 2014 |
| SCcd16 | II | 3 | MF196905 | China | 2016 |
| SH/CH/2016 | II | 3 | KY495781 | China | 2016 |
| JX/CH/2016 | II | 3 | KY495780 | China | 2016 |
| HZL1501 | II | 8 | MF669721 | China | 2015 |
| GZgy17 | II | 3 | MK144542 | China | 2017 |
| Em2007 | II | 8 | EU262603 | China | 2007 |
| QH-1(sh)/2008 | II | 8 | KT033733 | China | 2008 |
| PRRSV2/CN/101805/2018 | II | 5 | MT416548 | China | 2018 |
| Clone20 | II | 5 | FJ899592 | China | 2003 |
| rV68 | II | 5 | EU360128 | China | no |
| rV63 | II | 5 | EU360129 | China | no |
| DY | II | 5 | JN864948 | China | 2007 |
| SWU/CQ1/2018 | II | 5 | MK429987 | China | 2018 |
| FJSD | II | 5 | KP998474 | China | no |
| APRRS | II | 8 | GQ330474 | China | no |
| GD-KP | II | 3 | KU978619 | China | 2015 |
| SD110-1608 | II | 3 | MK780825 | China | 2016 |
| SDWH27-1710 | II | 3 | MK780824 | China | 2017 |
| SCya18 | II | 3 | MK144543 | China | 2018 |
| FJLIUY-2017 | II | 3 | MG011718 | China | 2017 |
| FJDJQ-2017 | II | 3 | MG011719 | China | 2017 |
| SDZC-1609 | II | 8 | MH651747 | China | 2016 |
| SD99-1606 | II | 1 | MH651745 | China | 2016 |
| FJZ03 | II | 1 | KP860909 | China | no |
| FJY04 | II | 1 | KP860910 | China | no |
| HENAN-XINX | II | 1 | KF611905 | China | 2013 |
| PRRSV2/CN/X4839/2017 | II | 1 | MT409692 | China | 2017 |
| CY2-1604 | II | 1 | MH651737 | China | 2016 |
| JSWA | II | 1 | KY373214 | China | 2014 |
| SDQZ-1609 | II | 1 | MH651746 | China | 2016 |
| SWU/MS3/2018 | II | 1 | MK429981 | China | 2018 |
| SWU/MS2/2018 | II | 1 | MK429980 | China | 2018 |
| SWU/YB1/2018 | II | 1 | MK429984 | China | 2018 |
| SWU/CD1/2018 | II | 1 | MK429986 | China | 2018 |
| SWU/YB2/2018 | II | 1 | MK429985 | China | 2018 |
| GXNN1839 | II | 1 | MN660070 | China | 2018 |
| SWU/MY6/2018 | II | 1 | MK429983 | China | 2018 |
| SWU/MY5/2018 | II | 1 | MK429982 | China | 2018 |
| SD53-1603 | II | 1 | MH651744 | China | 2016 |
| QHD1 | II | 1 | MG687491 | China | 2017 |
| NADC30 | II | 1 | MH500776 | China | 2017 |
| 10-10BJ-3 | II | 8 | JQ663542 | China | 2010 |
| HNjz15 | II | 1 | KT945017 | China | 2015 |
| HENXC-4 | II | 1 | KU950371 | China | 2015 |
| HENAN-HEB | II | 1 | KJ143621 | China | 2012 |
| CHsx1401 | II | 1 | KP861625 | China | 2014 |
| PRRSV2/CN/X4831/2018 | II | 1 | MT409687 | China | 2018 |
| FJ1402 | II | 1 | KX169191 | China | 2014 |
| PRRSV2/CN/X9830/2018 | II | 1 | MT409688 | China | 2018 |
| PRRSV2/CN/X4833/2018 | II | 1 | MT409691 | China | 2018 |
| FJL15 | II | 1 | KY412887 | China | 2014 |
| LNCH-1604 | II | 1 | MH651741 | China | 2016 |
| QHD2 | II | 1 | MH167387 | China | 2017 |
| PRRSV2/CN/110713/2018 | II | 1 | MT416546 | China | 2017 |
| TJZH-1607 | II | 1 | MH651748 | China | 2016 |
| HB17A | II | 1 | MG844181 | China | 2017 |
| HNJYH-1606 | II | 1 | MH651740 | China | 2016 |
| HNJYF-1606 | II | 1 | MH651738 | China | 2016 |
| HBFL-1604 | II | 1 | MH651739 | China | 2016 |
| QHD3 | II | 1 | MH167388 | China | 2017 |
| CH-WH-2019-1 | II | 1 | MK450333 | China | 2018 |
| SDQD-1604 | II | 1 | MH651742 | China | 2016 |
| HENXX-8 | II | 1 | KY041782 | China | 2016 |
| SD-1602 | II | 1 | MH651743 | China | 2016 |
| HENZMD-9 | II | 1 | KU950374 | China | 2015 |
| HNhx | II | 1 | KX766379 | China | 2016 |
| SCnj16 | II | 1 | MF196906 | China | 2016 |
| SC/DJY | II | 1 | MT075480 | China | 2019 |
| HENJY-2 | II | 1 | KX900392 | China | 2015 |
| FJDJQ-2018 | II | 1 | MT416547 | China | 2018 |
| FJDJQ-2018 | II | 1 | MN862433 | China | 2018 |
| PRRSV2/CN/X4836/2018 | II | 1 | MT409689 | China | 2018 |
| FJWQ16 | II | 1 | KX758249 | China | no |
| JL580 | II | 1 | KR706343 | China | 2013 |
| PRRSV2/CN/X2998/2018 | II | 8 | MT409690 | China | 2018 |
| SCN17 | II | 8 | MH078490 | China | 2017 |
| HLJZD30-1902 | II | 1 | MN648055 | China | 2019 |
| PRRSV-ZDXYL-China-2018-1 | II | 1 | MK453049 | China | 2018 |
| PRRSV-ZDXYL-China-2018-2 | II | 1 | MK453050 | China | 2018 |
| HLHDZD32-1901 | II | 1 | MN648449 | China | 2019 |
| HLJZD22-1812 | II | 1 | MN648450 | China | 2018 |
| LNDZD10-1806 | II | 1 | MN648054 | China | 2018 |

**Supplementary Table S2 The detailed information of clinical samples in this study**

| City | Collection year | Blood sample size | Serum sample size | Tissue sample size |
| --- | --- | --- | --- | --- |
| Chaozhou | 2022 | 3 | 0 | 0 |
| Foshan | 2022 | 0 | 2 | 0 |
| Guangzhou | 2022 | 0 | 0 | 1 |
| Heyuan | 2022 | 7 | 0 | 13 |
|  | 2023 | 0 | 60 | 0 |
| Jiangmen | 2022 | 15 | 0 | 9 |
|  | 2023 | 0 | 13 | 12 |
| Maoming | 2022 | 5 | 0 | 8 |
| Meizhou | 2022 | 0 | 0 | 5 |
| Qingyuan | 2021 | 8 | 0 | 0 |
|  | 2022 | 1 | 3 | 12 |
|  | 2023 | 1 | 0 | 0 |
| Shantou | 2022 | 2 | 0 | 2 |
| Shanwei | 2022 | 0 | 0 | 11 |
|  | 2023 | 9 | 0 | 0 |
| Shaoguan | 2022 | 0 | 0 | 2 |
| Yangjiang | 2022 | 27 | 8 | 11 |
|  | 2023 | 0 | 13 | 0 |
| Yunfu | 2022 | 0 | 0 | 51 |
| Zhaoqing | 2022 | 0 | 0 | 2 |
| Total |  | 78 | 99 | 139 |

**Supplementary Table S3. The information of reference sequences used for phylogenetic analysis in this study**

| Strain name | Virus genotype | Lineage | GenBank Accession no. | Country | Collection year |
| --- | --- | --- | --- | --- | --- |
| HZ-31 | II | 5 | KC445138 | China | 2012 |
| YN-2011 | II | 5 | JX857698 | China | 2011 |
| SD1-100 | II | 5 | GQ914997 | China | no |
| GS2002 | II | 5 | EU880441 | China | no |
| HNyc15 | II | 3 | KT945018 | China | 2015 |
| FJFS | II | 3 | KP998476 | China | no |
| QY2010 | II | 3 | JQ743666 | China | 2010 |
| GM2 | II | 3 | JN662424 | China | 2011 |
| FJXS15 | II | 1 | KX758250 | China | no |
| FJ1402 | II | 1 | KX169191 | China | 2014 |
| HENXC-4 | II | 1 | KU950371 | China | 2015 |
| JL580 | II | 1 | KR706343 | China | 2013 |
| HLJA1 | II | 8 | KT351739 | China | 2013 |
| MY-376 | II | 8 | KJ609517 | China | 2013 |
| HeNan-A1 | II | 8 | KJ002451 | China | 2013 |
| SDA2 | II | 8 | JX878379 | China | 2011 |
| LV | I | no | M96262 | no | no |
| HLJB1 | II | 8 | KT351740 | China | 2013 |
| NMEU09-1 | I | no | GU047345 | China | 2009 |
| BJEU06-1 | I | no | GU047344 | China | 2006 |
| SHE | I | no | GQ461593 | China | no |
| HH08 | II | 8 | JX679179 | China | 2011 |
| CH2002 | II | 8 | EU880438 | China | no |
| CH-1R | II | 8 | EU807840 | China | no |
| RFLP 1-4-4 lineage 1C variant | II | 1 | MW887655 | America | 2020 |
| IA/2014/NADC34 | II | 1 | MF326985 | America | 2014 |
| JXA1 | II | 8 | EF112445 | China | 2006 |
| QYYZ | II | 3 | JQ308798 | China | 2011 |
| VR-2332 | II | 5 | AY150564 | no | no |
| NADC30 | II | 1 | JN654459 | America | 2008 |
| HK4 | II | 8 | KF287134 | China | 2003 |
| Yunnan-08 | II | 8 | EU819086 | China | 2008 |
| HeN-2 | II | 8 | FJ237419 | China | 2007 |
| GXLSN06-2012 | II | 8 | KC618172 | China | 2012 |
| HBJM2 | II | 8 | EU399826 | China | 2007 |
| CH-1a | II | 8 | AY032626 | China | 1995 |
| HB-2(sh)/2002 | II | 8 | AY262352 | China | 2001 |

**Supplementary Table S4 Optimization of annealing temperature of duplex qPCR assay of NL-PRRSV and HP-PRRSV**

| Annealing temperature | Template | CT value | | | | | Amplification efficiency (%) |
| --- | --- | --- | --- | --- | --- | --- | --- |
|  |  | 1 × 10^6^ copies/μL | 1 × 10^5^ copies/μL | 1 × 10^4^ copies/μL | 1 × 10^3^ copies/μL | 1 × 10^2^ copies/μL |  |
| 50℃ | NL-PRRSV | 16.92 | 20.12 | 23.46 | 27.06 | 31.10 | 92.00 |
|  | HP-PRRSV | 15.21 | 18.60 | 21.92 | 25.15 | 28.78 | 98.06 |
| 55℃ | NL-PRRSV | 15.22 | 18.38 | 21.45 | 24.97 | 29.16 | 95.03 |
|  | HP-PRRSV | 15.54 | 20.45 | 23.59 | 26.19 | 29.38 | 99.16 |
| 60℃ | NL-PRRSV | 17.06 | 20.53 | 24.11 | 27.88 | 32.39 | 83.27 |
|  | HP-PRRSV | 14.81 | 18.83 | 23.04 | 26.26 | 29.75 | 85.35 |

**Supplementary Table S5 Optimization of annealing temperature of duplex qPCR assay of PRRSV-1 and PRRSV-2**

| Annealing temperature | Template | CT value | | | | | Amplification efficiency (%) |
| --- | --- | --- | --- | --- | --- | --- | --- |
|  |  | 1 × 10^6^ copies/μL | 1 × 10^5^ copies/μL | 1 × 10^4^ copies/μL | 1 × 10^3^ copies/μL | 1 × 10^2^ copies/μL |  |
| 50℃ | PRRSV-1 | 20.13 | 23.54 | 27.11 | 30.61 | 33.20 | 100.03 |
|  | PRRSV-2 | 16.10 | 19.55 | 22.90 | 26.64 | 29.40 | 98.06 |
| 55℃ | PRRSV-1 | 19.69 | 23.18 | 26.47 | 29.50 | 32.64 | 104.36 |
|  | PRRSV-2 | 15.12 | 18.27 | 21.80 | 25.31 | 28.68 | 96.20 |
| 60℃ | PRRSV-1 | 19.82 | 23.12 | 26.32 | 29.60 | 33.71 | 95.84 |
|  | PRRSV-2 | 14.54 | 17.70 | 20.91 | 24.35 | 27.29 | 104.64 |

**Supplementary Table S6 The consistency test results of specificity between this method and commercially available reagent kits**

| Template | VetMAX PRRSV EU&NA 2.0 Kit | | | |
| --- | --- | --- | --- | --- |
|  |  | Positive | Negative | agreement rate |
| PRRSV-1 | Positive | 22 | 0 | 95.8% |
|  | Negative | 2 | 24 |  |
| PRRSV-2 | Positive | 23 | 0 | 97.9% |
|  | Negative | 1 | 24 |  |
| HP-PRRSV | Positive | 24 | 0 | 100.0% |
|  | Negative | 0 | 24 |  |
| NL-PRRSV | Positive | 24 | 0 | 100.0% |
|  | Negative | 0 | 24 |  |
| PEDV | Positive | 0 | 0 | 100.0% |
|  | Negative | 0 | 48 |  |
| TGEV | Positive | 0 | 0 | 100.0% |
|  | Negative | 0 | 48 |  |
| PRV | Positive | 0 | 0 | 100.0% |
|  | Negative | 0 | 48 |  |
| PCV-2 | Positive | 0 | 0 | 100.0% |
|  | Negative | 0 | 48 |  |
| CSFV | Positive | 0 | 0 | 100.0% |
|  | Negative | 0 | 48 |  |
